# Development of a single-tube nested PCR-lateral flow biosensor assay for rapid and accurate detection of *Alternaria panax* Whetz

**DOI:** 10.1371/journal.pone.0206462

**Published:** 2018-11-08

**Authors:** Shuqin Wei, Yajuan Sun, Guangsheng Xi, Huijuan Zhang, Mingya Xiao, Rui Yin

**Affiliations:** 1 College of Agronomy, Jilin College of Agricultural Science and Technology, Jilin, Jilin, China; 2 Department of Neurology, China-Japan Union Hospital of Jilin University, Changchun, Jilin, China; 3 College of Traditional Chinese Medicine, Jilin College of Agricultural Science and Technology, Jilin, Jilin, China; 4 College of Biological and Pharmaceutical Engineering, Jilin College of Agricultural Science and Technology, Jilin, Jilin, China; University of Helsinki, FINLAND

## Abstract

*Alternaria panax* Whetz causes one of the most commonly occurring and serious diseases in ginseng cultivation, and may cause significant production and economic losses in the ginseng industry. Rapid, early, and accurate identification of *Alternaria panax* Whetz is an essential prerequisite for the effective prevention and control of further infection spread. In this work, a rapid and accurate molecular diagnostic method, a single-tube nested PCR-lateral flow biosensor assay (STNPCR-LFBA), was developed for rapid identification of *Alternaria panax* Whetz. The STNPCR-LFBA was 100 times more sensitive than the traditional PCR-LFBA. Besides that, the PCR product was checked by a lateral flow biosensor assay, which provided a basis for the migration of the detection technology to a point-of-care test (POCT) format. STNPCR-LFBA was specific to *Alternaria panax* Whetz, and no cross-reactions were observed in other non-target samples; the limit of detection was up to 0.01 pg of *Alternaria panax* Whetz genomic DNA. STNPCR-LFBA could also be used for specific identification of *Alternaria panax* Whetz in real samples. STNPCR-LFBA is useful for identifying *Alternaria panax* Whetz due to its rapidity, accuracy, and simple manipulation.

## Introduction

Ginseng, including Asian ginseng (*Panax ginseng* C. A. Meyer) and American ginseng (P. *quinquefolius* L.), is a highly valued traditional medicinal plant in Asia and North America. For thousands of years, ginseng has been widely used as a traditional medicine to improve immune and metabolic systems, enhance physical performance, and boost general vitality [1–5]. However, the plant easily contracts numerous diseases and is affected by the invasion of various pathogens due to its long growth period, which threatens the continued production of ginseng. *Alternaria panax* Whetz causes one of the most commonly occurring and serious diseases in ginseng cultivation, with an incidence rate of 20–30% that can increase to 100%; especially, more serious invasions could be induced in ginseng fields cultivated for more than 3 years [6].

The prevention and control of ginseng diseases mainly includes agricultural control, chemical control, and biological control, among which chemical control is widely used because of its high efficiency, quick effect, convenient operation, wide adaptability, and significant economic benefits [7]. However, a series of problems such as pesticide residues, pathogen resistance, environment pollution, and damage to natural enemies have emerged with the extensive use of chemical pesticides. Various studies have been conducted to solve these kinds of serious problems in ginseng disease with chemical control methods, but most of the main research directions have been focused on the separation and diagnosis of ginseng disease pathogens [8–11], screening of antagonistic bacteria for ginseng disease [12, 13] and detection of safe drugs to use [14–17]. There are few studies on the early diagnosis of the infection period before the onset of ginseng disease.

The traditional method of plant disease detection is mainly based on symptoms of diseased tissues, characteristics of pathogens, and the experience of investigators. However, it was difficult to accurately determine the disease because of similar symptoms caused by some pathogens with different symptoms only occurring at different times of onset. In the laboratory diagnosis of these diseases, isolation culture is the most common and widely utilized technique for the routine identification of plant pathogens [9–10]. However, isolation culture is time consuming and low accuracy due to the simultaneous presence of saprophytic bacteria.

In recent years, several DNA-based assays such as DNA probes, PCR, and nested PCR have been developed for the identification of plant pathogens. Recently, nested PCR has been widely used for the rapid, specific, and sensitive detection of various pathogens [18–20]. The nested PCR assay is a two-step amplification procedure that is carried out by amplifying the target fragment with the external primers and then using the PCR product as a template for amplification using the internal primers. Using this format, the sensitivity and specificity of test have increased, but there are still some drawbacks such as a labor-intensiveness, time-consumption, and the increased risk of carryover contamination. Researchers have attempted to establish single-tube nested PCR approaches, in which both external and inner primers are putted into a reaction tube, the annealing temperatures for initial PCR cycles are higher than the later PCR cycles. This single tube nested PCR reaction technology eliminating cross-contamination and maintaining high specificity and sensitivity [21–25]. However, this technique has been most applied for end-point gel electrophoresis detection or for expensive real-time fluorescence detection, thus hindered its routine use.

**Fig 1:**
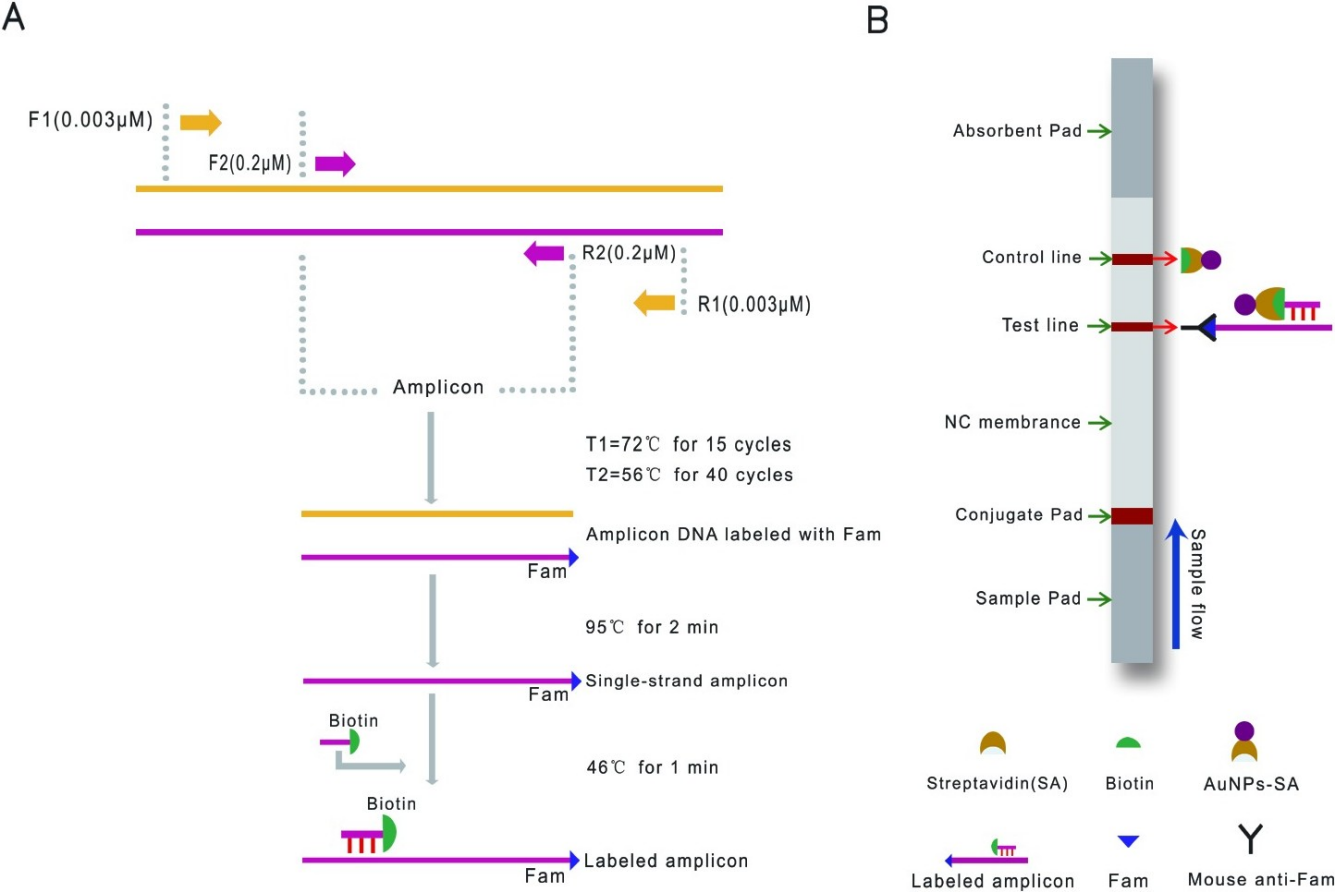
Schematic overview of the STNPCR-LFBA principle for detecting *Alternaria panax* Whetz. (A) Using the target DNA template, PCR amplification using the external primer pair (F_1_ and R_1_) will generate a PCR product that will be used for amplification by the inner primer pair (F_2_ and R_2_) in a single tube. The labeled single-stranded PCR product is hybridized with the labeled probe to form a nucleic acid hybridization complex (labeled with Biotin and Fam). (B) After placing the solution of the hybridization complex onto the sample pad of the biosensor, driven by the running stream, this complex is bound to a colloidal gold particle-streptavidin particles (AuNPs-SA) on the conjugate pad of the biosensor, followed by migrated further with the buffer stream, and the sample is trapped by the mouse anti-Fam antibody on the test line (T line) of the biosensor, the T line turns red. The excess unbound AuNPs-SA migrated and it can be captured by biotin on the control line (C line). The result is positive when both the T and C lines are red. The result is negative if only the C line is red.

In this work, we developed a highly specific and sensitive molecular technology that combines the single-tube nested PCR and a lateral flow biosensor assay for the identification of *Alternaria panax* Whetz, targeting the 18S ribosomal RNA sequence of *Alternaria panax* Whetz, and consists of an uninterrupted PCR in a single reaction tube. The single-strain PCR product was hybridized to a probe to form a nucleic acid hybridization complex (labeled with Biotin and Fam) (Fig 1A). The hybridization complex was then captured on the test line of the biosensor, and the test line was turned red and the result can be read by the naked eye within 2–3 minutes (Fig 1B). This accurate and easy-to-use system is ideal for further development as a point-of-care test.

## Materials and methods

### Materials and chemicals

*Alternaria panax* Whetz, *Cylindrocarpon destructans*, *Botrytis cinerea* Pers, *Sclerotionia schinseng*, *Phytophora cactorum* Schroet, and *Escherichia coli* were provided by the Plant Pathology Laboratory of Jilin Agricultural University. PCR Master Mix kit (ABI USA); Proteinase K; EDTA, Tris-HCl, NaCl, magnetic nanoparticles, isopropanol, 70% ethanol, and BSA-Biotin conjugate (Sangon Biotech Co., Ltd, Shanghai, China); 30 nm colloidal gold particles, gold absorbent pad (GL0194), and nitrocellulose filter membrane (Millipore 135) (Jiyi Biotech Co., Ltd, Shanghai, China); mouse anti-Fam antibody (Gei Man Biotech Co., Ltd, Guangzhou, China); streptavidin (Ruijin Biotech Co., Ltd, Shanghai, China), biosensor reader instrument (JY1501GS, Jie Yi Biotech Co., Ltd, Shanghai, China).

### DNA extraction

The *Alternaria panax* Whetz was inoculated on PDA media (20g potato, 2g glucose, 2g agar powder, 100 mL water), cultured at 25°C; *Alternaria panax* Whetz cells were harvested and re-suspended in 200 μL of TE buffer (Tris-EDTA), and then transferred to 1.5 mL centrifuge tubes; 600 μL of lysate buffer (100 mmol/L of EDTA, 100 mmol/L of Tris-HCl, 1.5 mol/L of NaCl, 1% SDS, pH 8.0) was added to the tube, 100 μL of 5 M guanidine hydrochloride lysis solution and 30 μL of ProteinaseK (20 mg/mL) was added, and incubated for 15 min at 65°C. The mixture was shaken 2–3 times, centrifuged at 12,000 rpm for 5 min, and the supernatant was transferred to another clean centrifuge tube, followed by addition of 600 μL of binding buffer (isopropanol) and 30μL of magnetic nanoparticles, The suspension was mixed by inversion and allowed to keep for 1 min at room temperature. The magnetic pellet was immobilized by application of an external magnet and the supernatant was removed. The magnetic pellet was washed with 70% ethanol twice and then dried thoroughly. The pellet was then completely re-suspended in 200μL of TE buffer (10 mM Tris-HCl, 1 mM EDTA, pH 8.0) and magnetic particle bound DNAs were eluted by incubation at 65°C with continuous agitation. The DNA was stored at -20°C and its concentration was determined by measuring the absorbance. Other purified DNA samples from five non-target pathogens including *Cylindrocarpon destructans*, *Botrytis cinerea* Pers, *Sclerotionia schinseng*, *Phytophora cactorum Schroet*, *and Escherichia coli* were also used. Each DNA sample was extracted and purified as described above. For DNA extraction from soil samples, 250 mg of the soil sample was added to 1 mL of lysate buffer, mixed completely, and DNA was then extracted as described above.

### Design of primers and probes and PCR conditions

The 18S ribosomal RNA sequence of *Alternaria panax Whetz* (accession number: FJ607183) was searched in GenBank. To increase the specificity of PCR, an oligonucleotide probe that can be hybridized to the PCR product was used in this study, the primers and gene-specific probe were designed using DNAStar software. Primer and probe specificity were verified by the BLASTn program in GenBank (http://www.ncbi.nlm.nih.gov/BLAST). The primer and probe sequences used in the present study are listed in Table 1. The primers and probes were synthesized by Sangon Biotech Co., Ltd, Shanghai, China.

**Table 1:** Primers and probes.

PCR types	Primer/Probe names	Sequence (5’-3’)	TM (°C)
Traditional PCR	F_2_	gctggccttgctgaat	56
	R_2_	Fam-gtgcgttcaaagattcg	56
	P	gtcagtaacaacataa-Biotin	45
Single-tube nested PCR	F_1_	gaacctgcggagggatcattacac	72
	R_1_	gtggacgctgaccttggctggaag	72
	F_2_	gctggccttgctgaat	56
	R_2_	Fam-gtgcgttcaaagattcg	56
	P	gtcagtaacaacataa-Biotin	45

Traditional PCR and single-tube nested PCR were performed using a standard system (Applied Biosystems, Foster City, CA, USA). For the PCR, a typical reaction mix was prepared with 15 μL of PCR master mix (Qiagen, USA), 1 μL of genomic DNA, 1 μL of each primer, and ddH_2_O to a total volume of 25 μL. For the traditional PCR, the amplification conditions were as follows: 95°C for 2 minutes; followed by 40 cycles of 95°C for 30 seconds, 56°C for 30 seconds, 72°C for 30 seconds; and 95°C for 2 minute, after which the PCR product was placed on ice. For single-tube nested PCR, the conditions were 95°C for 2 minutes; 15 cycles of denaturation at 95°C for 30 seconds and annealing at 72°C for 30 seconds; 40 cycles of denaturation at 95°C for 30 seconds, annealing at 56°C for 30 seconds, and extension at 72°C for 30 seconds; and 95°C for 2 minute, after which the PCR product was placed on ice.

### Construction of the lateral flow biosensor

The lateral flow biosensor was constructed as described by Yin [26] with some modifications. Colloidal gold particles-streptavidin conjugate (AuNPs-SA) was prepared by conjugating 6.5 mg/mL of streptavidin (SA) to 30 nm colloidal gold nanoparticles (AuNPs), and this was impregnated on the gold nanoparticle conjugate pad of the biosensor. Mouse anti-Fam antibody and BSA-Biotin conjugate were coated on the test line (T line) and control line (C line) of the nitrocellulose filter membrane (NC), respectively, the concentration of the mouse anti-Fam antibody and BSA-Biotin conjugate was 1.0 mg/mL. The biosensor was assembled with a lower absorbent pad, a gold nanoparticle conjugate pad, an NC membrane, and an upper absorbent pad. The card was then cut into 0.5 cm wide, 5 cm long pieces. The biosensors were sealed in a plastic bag and stored at room temperature until use.

### Lateral flow biosensor protocol

For the lateral flow biosensor assay, 10 μL of the denatured PCR product and 1 μL of the gene-specific probe (10 μM) were added to 50 μL of hybridization buffer [(4× SSC, 2% BSA), pH 7.0], mixed and then incubated at 46°C for 1 min. Next, 10 μL of the hybridization product was placed onto the sample pad of the biosensor, and the lower part of the biosensor was dipped into 100 μL of the sunning buffer (0.01 M PBS, pH 7.4), the result was read visually within 5 min. For quantitative measurements, the biosensor was placed into the biosensor reader, and the ratio of the T to the C line signals is proportional to the amount of the target molecule.

#### Optimization of hybridization conditions

To improve the sensitivity of the lateral flow biosensor assay, several reaction conditions were systematically investigated by comparing the performance of the lateral flow biosensor assay in detecting the target PCR amplicon, mainly including hybridization temperature, hybridization time, probe concentration, and composition of the hybridization buffer. To find the best temperature for hybridization, several hybridization temperatures, including 31°C, 36°C, 41°C, 46°C and 51°C were set on the heat block, and the hybridization was carried out for 5 min. The products were then detected by the lateral flow biosensor and the results were measured using the biosensor reader instrument. The probe concentrations, mainly including 15 μM, 10 μM, 5 μM, 0.5 μM, and 0.1 μM could only be investigated when the best hybridization temperature was determined. Based on the optimal hybridization conditions determined previously, different hybridization times (1 min, 5 min, 10 min and 15 min) were evaluated accordingly. Finally, the optimal composition of the hybridization buffer was also studied systematically, including the concentrations of SSC (2×, 4×, 6×, and 8×) and BSA (2%, 4%, 6%, and 8%).

### Electrophoresis of PCR products

Ten PCR products were electrophoresed on 2% agarose gel at 110V for 30 minutes, and the results were recorded using a gel imaging system.

### Evaluation of the newly developed assay

To assess the sensitivity of the assay, genomic DNA of the purified *Alternaria panax* Whetz was diluted serially 10-fold with ddH_2_O; next, 1 ng, 0.1 ng, 0.01 ng, 1 pg, 0.1 pg, 0.01pg, and 1 fg templates were prepared for the PCR. The PCR product was detected by the lateral flow biosensor assay. To investigate the specificity of the test, the *Alternaria panax* Whetz and other non-target ginseng pathogens (*Cylindrocarpon destructans*, *Botrytis cinerea* Pers *Sclerotionia schinseng*, *Phytophora cactorum* Schroet) which are commonly found in the soil cultivated ginseng were used in this study. Besides that, a bacterium (*Escherichia coli*), which has high genomic sequence homology with the primer, was also used. The genomic DNA of *Alternaria panax* Whetz, *Cylindrocarpon destructans*, *Botrytis cinerea* Pers, *Sclerotionia schinseng*, *Phytophora cactorum* Schroet, and *Escherichia coli* was extracted, and 1 ng of each genomic DNA sample was used as a template for single-tube nested PCR followed by detection using the lateral flow biosensor assay. To evaluate the practicability of the newly developed assay in the detection of real specimens, 12 soil samples from a cultivated ginseng field, 6 soil samples from uncultivated field of ginseng (a maize field) were collected and then tested by the STNPCR-LFBA. The evaluation of the specificity, sensitivity and the feasibility of the test on real soil samples were performed with replicates for three times.

## Results

### PCR for 18S rRNA sequence

**Fig 2:**
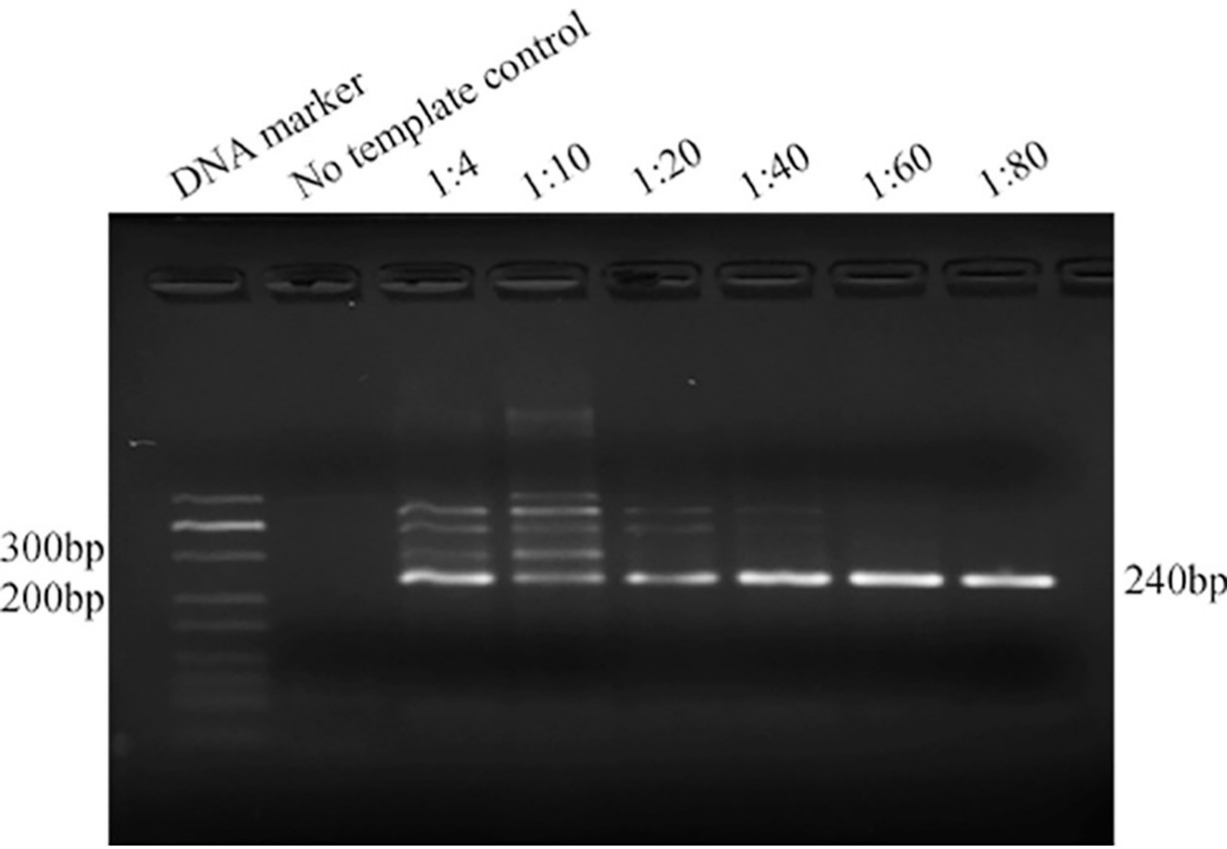
Optimization of the external/inner primer concentration. Lanes 1: DNA marker, lanes 2: no template control, lanes 3–8: ratios of external vs. inner primer (1: 4, 1: 10, 1: 20, 1: 40, 1:60, and 1: 80).

Using 1 ng of genomic DNA from *Alternaria panax* Whetz as the template, 475 bp and 240 bp fragments were amplified by the external primers F_1_/R_1_ and the inner primers F_2_/R_2_, respectively. These PCR amplicons were of the expected size and were confirmed by sequencing (data not shown). For single-tube nested PCR, the ratios of the outer primers to the inner primers, including 1 to 4, 1 to 10, 1 to 20, 1 to 40, 1 to 60, and 1 to 80 were also studied. All six tests generated the target band, but the most specific and strongest target band was generated when the ratio of the primers was 1 to 60 (Fig 2). Thus, the optimized concentrations of each external primer (F_1_/R_1_) and inner prime (F_2_/R_2_) were 0.003 μM and 0.2 μM, respectively.

### Optimization of the hybridization conditions

**Fig 3:**
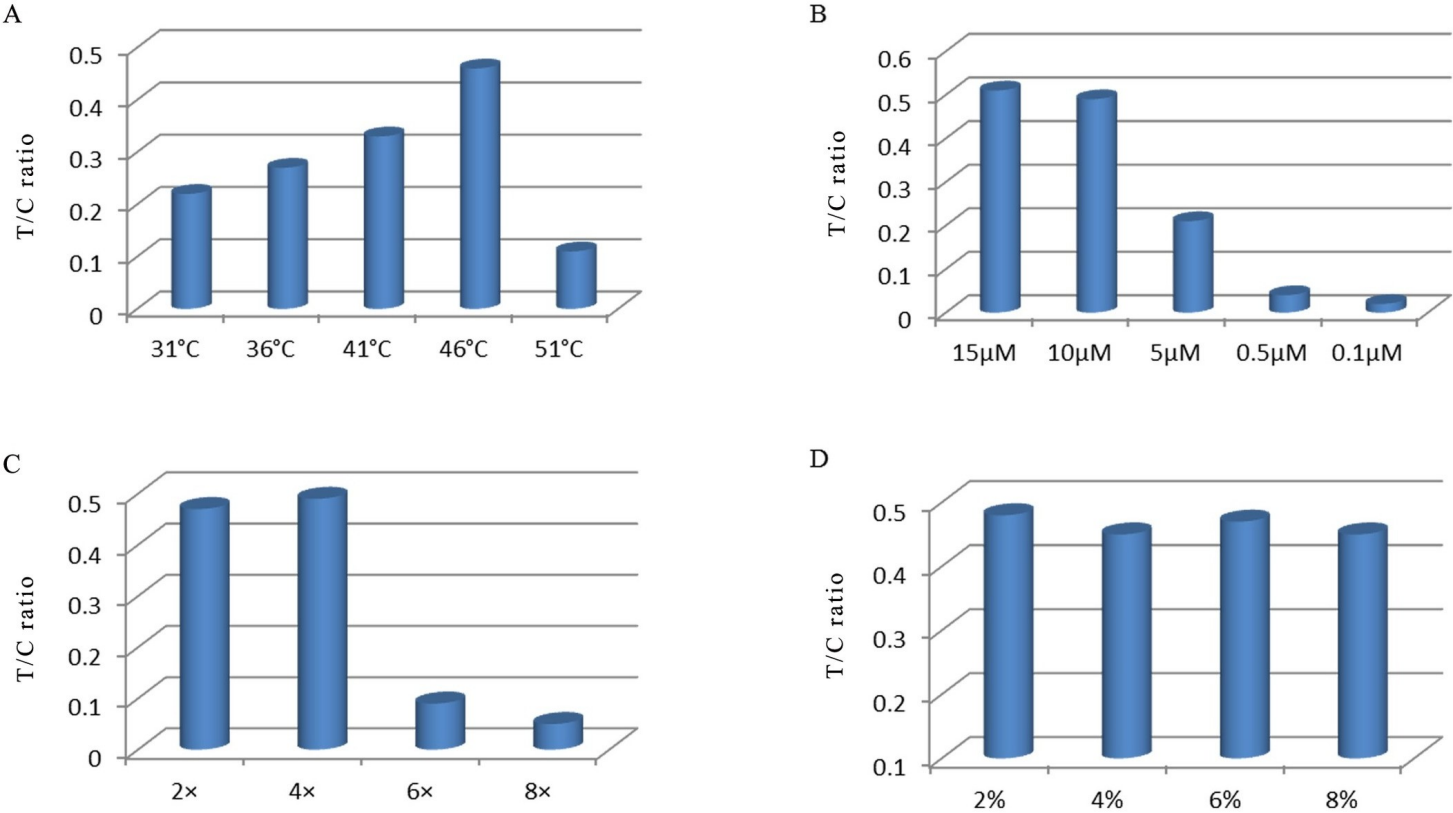
Optimization of hybridization conditions. (A) Effect of the hybridization temperatures on the T/C ratio of LFBA, (B) effect of the probe concentration on the T/C ratio of LFBA, (C) effect of the SSC concentration on the T/C ratio of LFBA and (D) effect of the BSA concentration on the T/C ratio of LFBA.

In the present study, there was a strongest the T/C ratio when the temperature was set at 46°C for the hybridization (Fig 3A). The probe concentration was 10 μM, and increasing its amount produced no obvious signal change in the T/C ratio (Fig 3B). The T/C ratios were not found to be increased with prolonged hybridization time; thus, 1 min was used for hybridization (data not shown). The optimal concentration of the hybridization buffer was 4× SSC matrix and 2% BSA (Fig 3C and 3D).

### Evaluation of the newly developed assay

**Fig 4:**
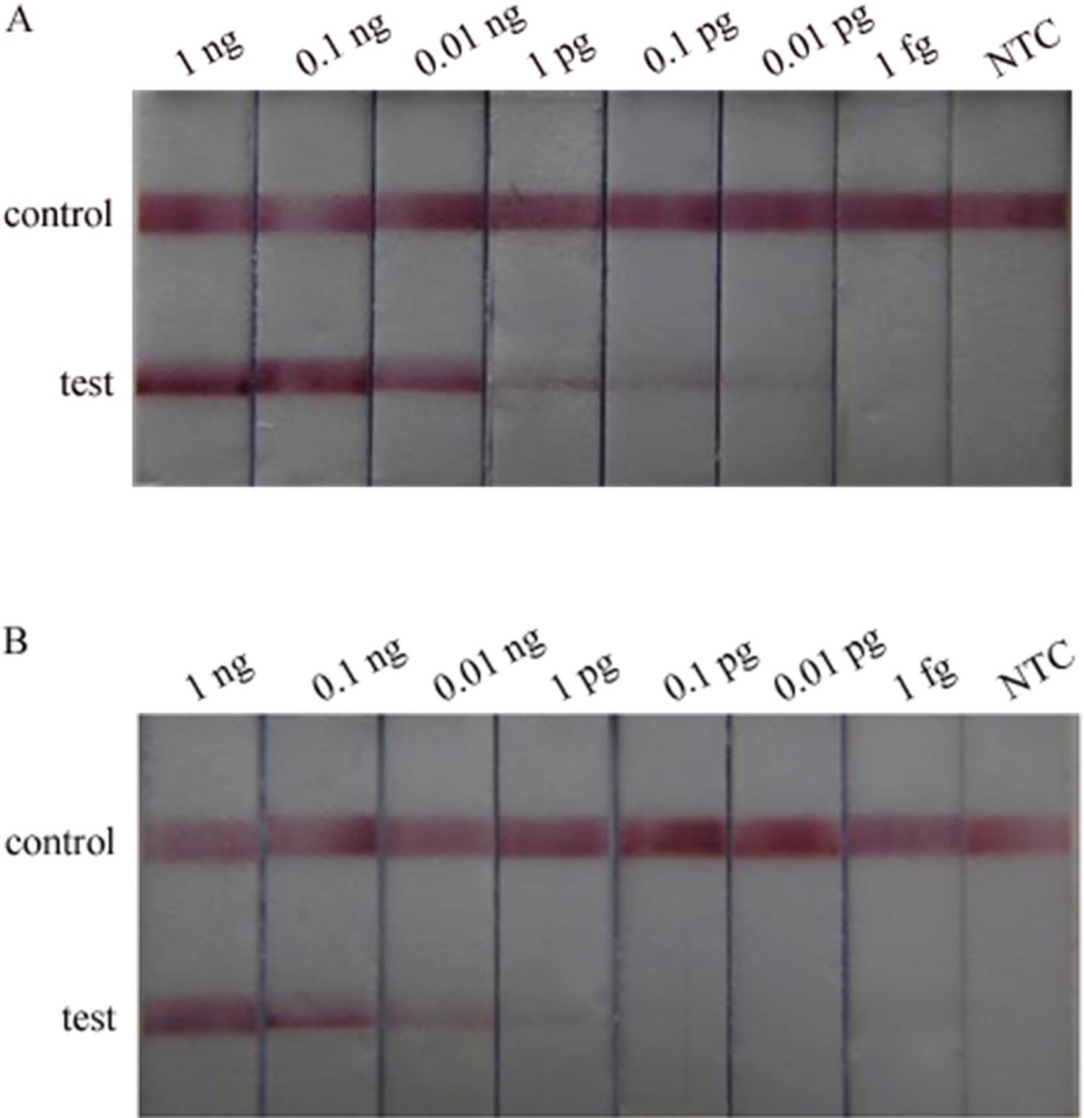
The sensitivity of the test. (A) STNPCR-LFBA and (B) TPCR-LFBA, NTC: no template control, the *Alternaria panax* Whetz DNA templates were serially diluted 10-fold from 1 ng to 1 fg.

The sensitivity of the single-tube nested PCR-lateral flow biosensor (STNPCR-LFBA) and the traditional PCR-lateral flow biosensor assay (TPCR-LFBA) was evaluated using serial 10-fold dilutions of *Alternaria panax* Whetz DNA. STNPCR-LFBA could identify 0.01pg of *Alternaria panax* Whetz DNA (Fig 4A), whereas TPCR-LFBA could only identify 1 pg of *Alternaria panax* Whetz DNA (Fig 4B). Thus, the STNPCR-LFBA was 100 times more sensitive than TPCR-LFBA.

**Fig 5:**
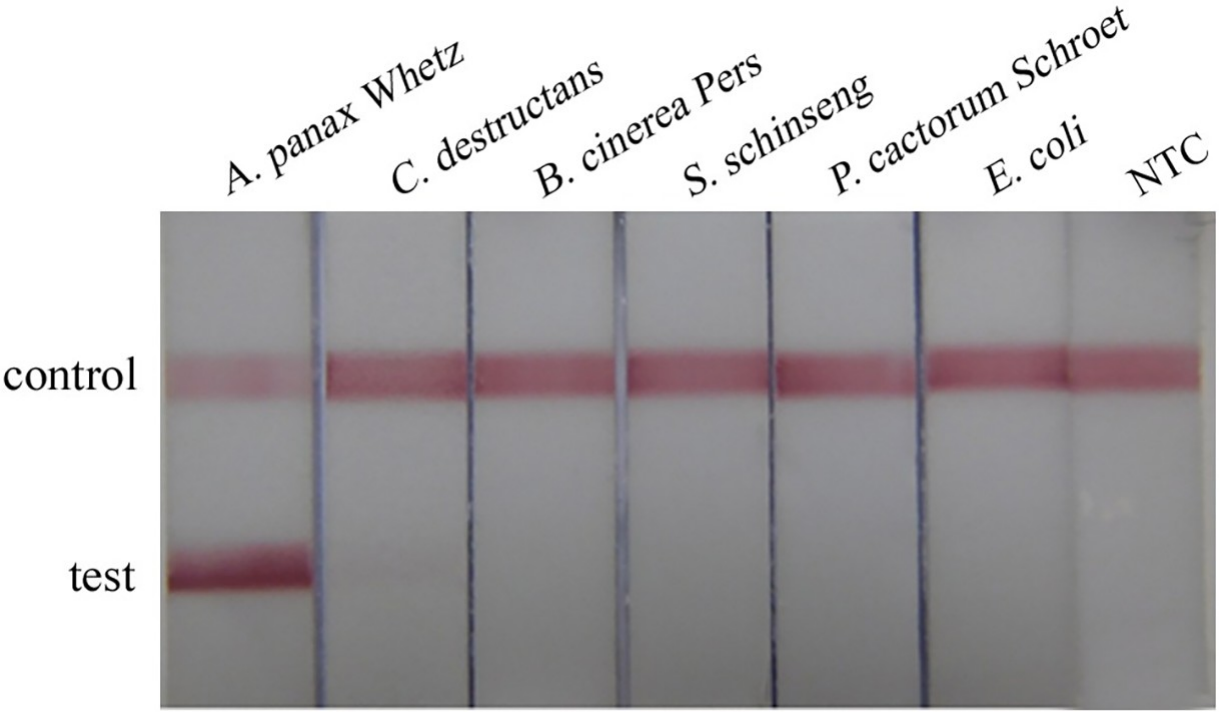
The specificity of STNPCR-LFBA. *A*. Whetz *Alternaria panax-Alternaria panax* Whetz, *C*. *destructans-Cylindrocarpon destructans*, *B*. *cinerea* Pers-*Botrytis cinerea* Pers, *S*. *schinseng-Sclerotionia schinseng*, *P*. *cactorum Schroet-Phytophora cactorum Schroet*, *E*. *coli-Escherichia coli*, NTC: no template control.

To evaluate the specificity of STNPCR-LFBA, the DNA of *Alternaria panax* Whetz and other non-target DNA samples were tested using STNPCR-LFBA. The result showed that only *Alternaria panax* Whetz DNA was detected as positive and no cross-reactions were found with other non-target DNA samples (Fig 5).

**Fig 6:**
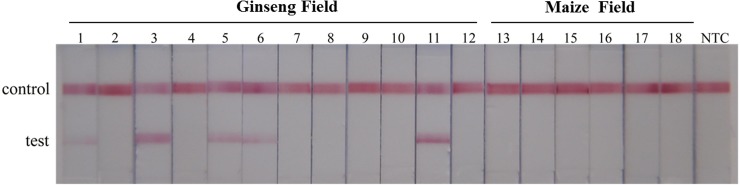
Performance of the STNPCR-LFBA on real samples. Biosensor 1–12: STNPCR-LFBA results for 12 soil samples cultivated ginseng, biosensor 13–18, STNPCR-LFBA results for 6 soil samples cultivated maize. NTC: no template control.

To evaluate the real application of the test, 18 soil samples were test by STNPCR-LFBA (Fig 6). 5 of 12 soil samples cultivated ginseng were positive, and all the positive PCR products were confirmed by sequencing (part of the sequencing results in S1 Fig). And after a long period of cultivation, several ginsengs cultivated near the positive soils were suffered from the black spot disease, which were highly concordant with the detection results in this study. In the control group, all the tests were negative, indicating that the specificity of the test was 100%.

## Discussion

*Alternaria panax* Whetz causes one of the most commonly occurring and serious diseases during ginseng cultivation, and may cause significant production and economic losses in the ginseng industry. Rapid, early, and accurate identification of *Alternaria panax* Whetz is an essential prerequisite for the effective prevention and control of further infection spread. The STNPCR-LFBA described here has been developed as a new molecular technology for simple, fast, and sensitive identification of *Alternaria panax* Whetz. STNPCR-LFBA combines both the specificity and sensitivity of single-tube nested PCR with the simplicity and speed of a lateral flow biosensor (visible after 2–3 min by the naked eye). Biosensors, in contrast to traditional detection technologies, do not rely on post amplification preparation or sophisticated equipment, and are especially suitable for POC use.

The reliability of STNPCR-LFBA was determined through detection multiple soil samples from the fields. Using the STNPCR-LFBA detection format, 5 of 12 soil samples cultivated ginseng were positive, whereas the control group was negative. These preliminary findings indicate that this method is reliable for practical application. In the next step, we will apply for early diagnosis of the pathogen before the onset of ginseng disease in large samples, and evaluate it value in the prevention and control of such disease in the field.

There are still several drawbacks that must be addressed in follow-up studies. In the present study, it is involved manually operation steps for the extraction of the DNA samples, which are not user friendly; moreover, it is still needed to open the tube after PCR reaction for detection, thus increases the risk of aerosol containment. It is imperative to establish a POCT detection technology that combines DNA extraction, PCR amplification and biosensor detection in an integrated and automatic detection platform. Once an integrated sample-in to answer-out detection system is established, this assay could eventually be used at POC settings.

## Conclusions

In this study, STNPCR-LFBA, a single-tube nested PCR combined with a lateral flow biosensor assay was established for the rapid and accurate identification of *Alternaria panax* Whetz. This single tube nested PCR technology is 100 times more sensitive than the traditional PCR; the PCR product is checked by a lateral flow format, and the result is visualized on the biosensor with no requirement of special equipment and trained technician. This rapid, sensitive, and easy-to-read format could have potential applications in plant disease monitoring, prevention, and control in the field, especially in low-equipment laboratories.

## Supporting information

S1 Fig The sequencing result of the PCR amplicon. (TIF)
