# Supplementary figures and images for: Development of a single-tube nested PCR-lateral flow biosensor assay for rapid and accurate detection of Alternaria panax Whetz
Source: PLoS One. 2018 Nov 8;13(11):e0206462. doi: 10.1371/journal.pone.0206462 (PMC6224276; doi:10.1371/journal.pone.0206462)

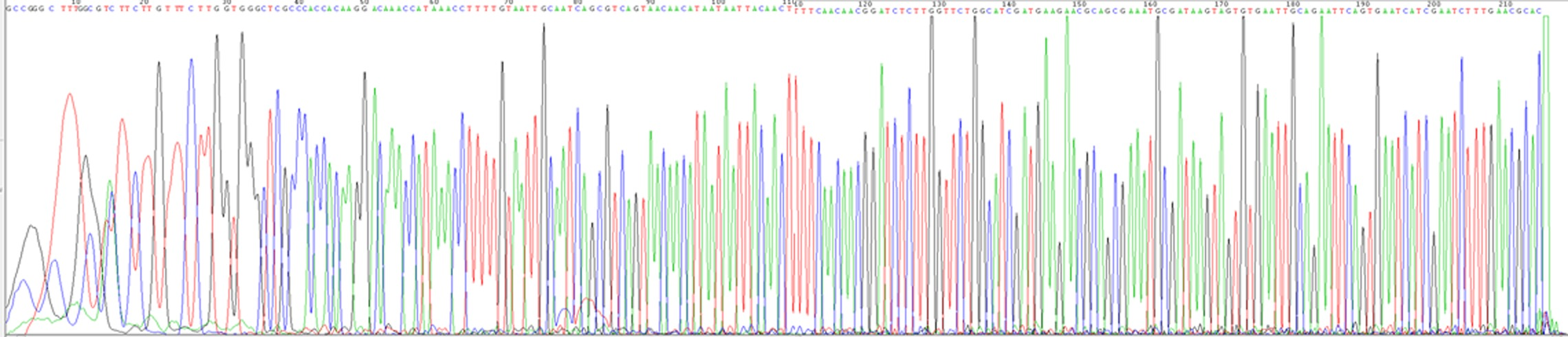

Supplement: S1 Fig — (TIF) [file pone.0206462.s001.tif]
